# Supplementary material for: Temporal Effects on Radiation Responses in Nonhuman Primates: Identification of Biofluid Small Molecule Signatures by Gas Chromatography–Mass Spectrometry Metabolomics
Source: Metabolites. 2019 May 15;9(5):98. doi: 10.3390/metabo9050098 (PMC6571779; doi:10.3390/metabo9050098)
Supplement: Supplementary file 1 [file metabolites-09-00098-s001.zip › Supplementary Materials/Supplementary Materials.pdf]

Table S1

Normalized abundance of compounds detected by GC-TOF-MS global profiling of NHP biofluids after 4 Gy γ radiation exposure.

	Metabolite name	Pre	1 d	3 d	5 d	7 d	15 d	21 d	28 d	60 d
Urine	Allantoic acid	0.14±0.05	0.10±0.04	0.12±0.07	0.02±0.01	0.00±0.00	0.01±0.01	0.02±0.02	0.00±0.00	0.02±0.02
	5-Hydroxyindoleacetic acid	0.01±0.00	0.02±0.01	0.01±0.00	0.00±0.00	0.00±0.00	0.02±0.01	0.02±0.01	0.00±0.00	0.04±0.01
Serum	Oleic acid	0.23±0.09	0.00±0.00	0.59±0.19	1.25±0.25	1.06±0.33	0.81±0.13	0.92±0.13	0.69±0.11	0.69±0.03
	Inosine	1.31±0.32	0.96±0.22	1.40±0.46	0.96±0.41	0.42±0.22	0.00±0.00	0.00±0.00	0.36±0.18	0.00±0.00
	Leucine	2.20±0.20	2.70±0.46	4.97±0.51	3.57±0.44	3.43±0.63	2.73±0.36	3.86±0.61	2.32±0.40	3.09±0.64
	Isoleucine	1.33±0.09	1.68±0.20	2.51±0.17	1.78±0.16	1.80±0.24	1.57±0.15	1.85±0.27	1.44±0.21	1.67±0.32
	Valine	0.23±0.09	0.00±0.00	0.59±0.19	1.25±0.25	1.06±0.33	0.81±0.13	0.92±0.13	0.69±0.11	0.69±0.03
	Serine	4.57±0.41	4.69±0.43	6.92±0.53	5.32±0.44	5.57±0.55	4.71±0.49	5.81±0.46	4.20±0.36	4.71±0.73
	Threonine	4.92±0.48	6.98±0.67	7.58±0.40	5.23±0.54	5.71±0.83	4.36±0.35	4.90±0.57	4.03±0.41	5.66±1.02
	Phenylalanine	2.45±0.17	3.10±0.27	3.38±0.33	2.60±0.24	2.83±0.23	2.72±0.17	3.26±0.36	1.99±0.18	2.51±0.19

(Mean ± SEM)

Table S2

Comparisons of log fold changes (logFC) of urinary TCA cycle intermediates at 7 d after a 4 Gy γ radiation exposure in a previous NHP cohort [25,26] and the current cohort.

**Biofluid**	**Metabolite**	**logFC Control vs. 7 d Previous Cohort**	**logFC Control vs. 7 d Current Cohort**
Urine	Citric acid	-0.35	-0.27
	Isocitric acid	-0.26	-0.20
	cis-Aconitic acid	-0.18	-0.08
	Malic acid	-0.11	-0.26
	Succinic acid	-0.13	-0.21
	Fumaric acid	0.05	-0.11

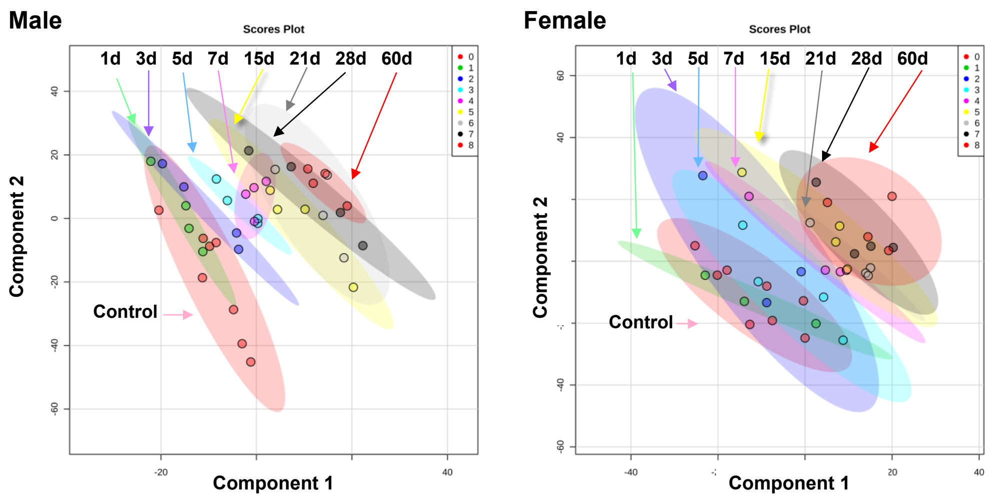

Figure S1. PLS-DA plots comparing pre-exposure to 1 – 60 d in urine for males and females after 4 Gy γ-ray TBI in NHPs. Similar separation is observed as grouped analysis, however, females show higher variation than males (graphs generated in MetaboAnalyst 4.0, pre-exposure samples [-8 and -3 d] were averaged for the control group).

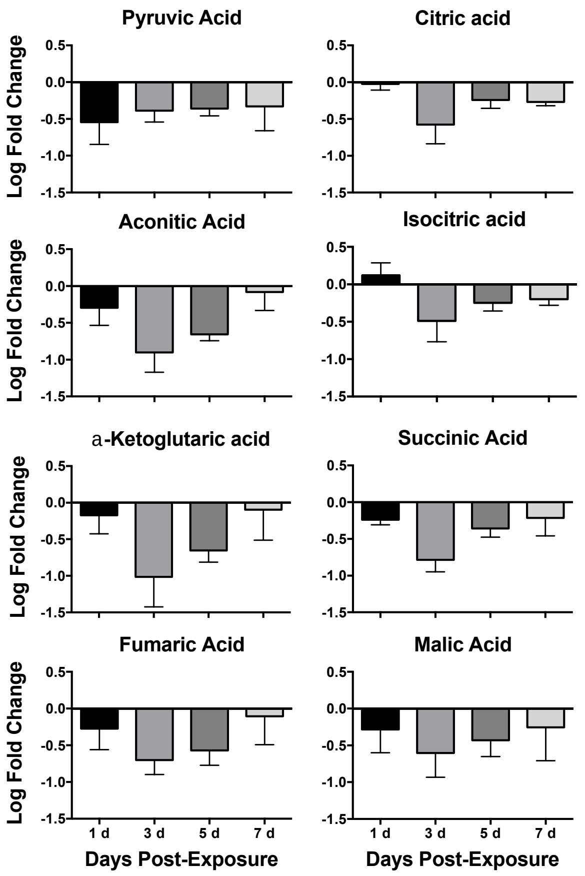

Figure S2. Log fold change of urinary TCA cycle intermediates significantly perturbed after 4 γ Gy TBI in NHPs from 1 – 7 d. (pyruvic acid [P=0.085], citric acid [P=0.027], isocitric acid [P=0.041], succinic acid [P=0.004], *cis*-aconitic acid [P=0.047], fumaric acid [P=0.131], malic acid [P=0.196], α-ketoglutaric acid [P=0.033]; P < 0.05 determined by a Kruskal-Wallis test, mean ± SEM, pre-exposure samples [-8 and -3 d] were averaged for the control group).
